# Alternative Polyadenylation and Salicylic Acid Modulate Root Responses to Low Nitrogen Availability

**DOI:** 10.3390/plants9020251

**Published:** 2020-02-16

**Authors:** Carlos M. Conesa, Angela Saez, Sara Navarro-Neila, Laura de Lorenzo, Arthur G. Hunt, Edgar B. Sepúlveda, Roberto Baigorri, Jose M. Garcia-Mina, Angel M. Zamarreño, Soledad Sacristán, Juan C. del Pozo

**Affiliations:** 1 Centro de Biotecnología y Genómica de Plantas (CBGP), Instituto Nacional de Investigación y Tecnología Agraria y Alimentaria (INIA), Campus de Montegancedo, Pozuelo de Alarcón, 28223 Madrid, Spain; cm.conesa@upm.es (C.M.C.); sara.navarro@inia.es (S.N.-N.); 2 Centro de Biotecnología y Genómica de Plantas (CBGP) and Escuela Técnica Superior de Ingeniería Agronómica, Agroambiental y de Biosistemas (ETSIAAB), Universidad Polictécnica de Madrid, Campus de Montegancedo, Pozuelo de Alarcón, 28223 Madrid, Spain; soledad.sacristan@upm.es; 3 DTD Development and Technical Department, Timac Agro Spain, 31580 Lodosa, Navarra, Spain; angela.saez@upm.es (A.S.); rbaigorri@timacagro.es (R.B.); 4 Department of Plant and Soil Sciences, University of Kentucky, Lexington, KY 40546-0312, USA; ldeloren@fiu.edu (L.d.L.); aghunt00@uky.edu (A.G.H.); 5 Departamento de Biotecnología y Bioingeniería CINVESTAV Instituto Politécnico Nacional, 07360 Ciudad de Mexico, Mexico; esepulveda@unpa.edu.mx; 6 Environmental Biology Department, University of Navarra, 31008 Navarra, Spain; jgmina@unav.es (J.M.G.-M.); angelmarizama@unav.es (A.M.Z.)

**Keywords:** root development, alternative polyadenylation, nitrogen starvation, salicylic acid

## Abstract

Nitrogen (N) is probably the most important macronutrient and its scarcity limits plant growth, development and fitness. N starvation response has been largely studied by transcriptomic analyses, but little is known about the role of alternative polyadenylation (APA) in such response. In this work, we show that N starvation modifies poly(A) usage in a large number of transcripts, some of them mediated by FIP1, a component of the polyadenylation machinery. Interestingly, the number of mRNAs isoforms with poly(A) tags located in protein-coding regions or 5′-UTRs significantly increases in response to N starvation. The set of genes affected by APA in response to N deficiency is enriched in N-metabolism, oxidation-reduction processes, response to stresses, and hormone responses, among others. A hormone profile analysis shows that the levels of salicylic acid (SA), a phytohormone that reduces nitrate accumulation and root growth, increase significantly upon N starvation. Meta-analyses of APA-affected and *fip1-2*-deregulated genes indicate a connection between the nitrogen starvation response and salicylic acid (SA) signaling. Genetic analyses show that SA may be important for preventing the overgrowth of the root system in low N environments. This work provides new insights on how plants interconnect different pathways, such as defense-related hormonal signaling and the regulation of genomic information by APA, to fine-tune the response to low N availability.

## 1. Introduction

Nitrogen (N) is a key mineral nutrient that plays a crucial role in plant growth and development. Although different N compounds can be found in soil, nitrate (NO_3_^−^) is the major N source for higher plants and one of the most assimilable N-based compound [1]. Root architecture system can be modified by nutrient availability, affecting root length, lateral roots (LRs) number, LRs emergence angle, root diameter, and root hair density and length [2]. The root system can be enlarged by the formation of LRs, which are lateral organs with a postembryonic development [3,4,5]. A morphological analysis in Arabidopsis showed that a moderate N deficiency increases the total root length while a severe N deficiency decreases total length [2]. Under low N conditions, to guarantee the search and influx of N into the roots, plants induce a large number of genes, many of them involved in N acquisition and N usage efficiency. To cope with N acquisition, high and low affinity NO_3_^−^ transport systems, HATS and LATS, respectively, have evolved during evolution [6]. The expression of one of these high affinity transporters, *NRT2.4*, is rapidly induced by N starvation [7], and therefore can be used as a N starvation marker in plants.

Gene transcription is probably the most important mechanism to regulate transcript accumulation. However, alternative splicing and/or polyadenylation of mRNAs provides an additional level of regulation that expands the transcriptome and, therefore, amplifies the coding capacity of the genome [8,9]. Differential selection of polyadenylation sites (alternative polyadenylation or APA) can also affect mRNA stability or the protein translation efficiency of transcripts [8,10,11]. Although polyadenylation typically occurs at the end of the 3′-UTR (and actually defines it), polyadenylation may also occur within introns, protein-coding regions, and 5′-UTRs, which are termed as non-canonical. The levels of mRNA isoforms with non-canonical polyadenylation increase in response to abiotic stresses [12,13,14], suggesting a role of these non-canonical mRNA isoforms during the plant response to these stresses. For example, in Arabidopsis, an increase in mRNA isoforms with 3′ ends in coding regions and 5′-UTRs is seen in response to hypoxia [14]. Similarly, elevated salt concentrations are accompanied by increased levels of non-canonical mRNA isoforms [12]. Interestingly, this elevated non-canonical polyadenylation is partially dependent on FIP1 (factor interacting with poly(A) polymerase 1) activity [12]. APA also seems to be important to maintain correct hormonal signaling, as in the case of the hormone auxin [15]. Recently, it was reported that polyadenylation plays an important role in nitrate (NO_3_^−^) signaling in Arabidopsis. Mutations in two components of poly(A) machinery, *FIP1* and *CPSF30*, affect the response of Arabidopsis seedlings to NO_3_^−^ signaling [16,17]. In both mutants, *fip1* and *cpsf30/ots6*, the up-regulation of nitrate-responsive genes after nitrate addition does not occur, suggesting that APA mediated by *FIP1* and *CPSF30* is needed for such regulation.

In this work, we have found that the *fip1-2* mutant, which has defects in polyadenylation [12], is less sensitive to the root growth inhibition promoted by N deficiency and has a lower induction of *NRT2.4* transporter. We have identified a large number of genes presenting differences in poly(A) usage, and similar to other abiotic stresses, N deprivation favors the non-canonical poly(A) usage, mainly in the 5′-UTR in a *FIP1*-dependent manner. Genes showing APA belong to different functional categories such as N-metabolism, and respond differently to stresses or hormonal signaling. Finally, by meta-analyses of APA-modified and *fip1-2*-deregulated genes we found that salicylic acid (SA) functions in the plant response to nitrogen starvation, likely by preventing the overgrowth of the root system in low N. Taken together, our data indicate that APA and SA accumulation function in the root responses to N deficiency.

## 2. Results

### 2.1. fip1-2 Mutant has an Altered N Starvation Response in Roots

**Figure 1:**
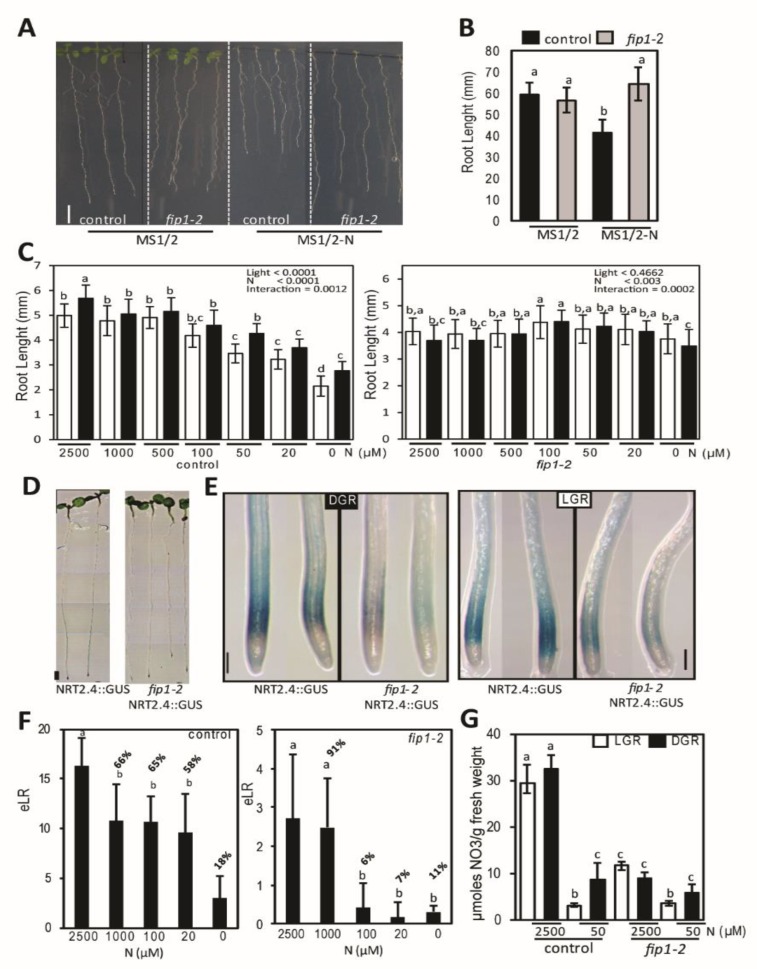
Root illumination and polyadenylation affect root growth and LR formation under nitrogen starvation. (**A**) Representative pictures of control and *fip1-2* mutant grown for 10 days in MS1/2 with or without nitrogen. Scale bar: 1cm. (**B**) Root growth quantification of seedlings grown as in A. (**C**) Root growth of 10-days-old control or *fip1-2* seedlings grown with different concentrations of nitrogen. White and black bars correspond to light- or dark-grown root seedlings respectively. (**D**) Representative picture of GUS staining NRT2.4::GUS seedlings grown in MS1/2 medium for 5 days and then transferred to MS1/2 containing 50 µM of N for 2 extra days. Scale bar: 1 cm. (**E**) Higher magnification of the root tip from seedlings shown as in A. Scale bar: 500 µm. (**F**) Number of emerged lateral roots (eLR) of control or *fip1-2* seedlings grown in MS medium containing different concentrations of nitrogen during 10 days. Numbers above the bar correspond to the percentage of eLR with respect to the maximum nitrogen medium (2500 µM). (**G**) Amount of NO_3_^−^ in control or *fip1-2* grown during 10 days in medium containing high N (2500 µM) or low N (50 µM). (B,F) Significance was analyzed by ANOVA and Tukey HSD post-hoc test. *p* < 0.05. (C,G) Significance was analyzed by two-way ANOVA and Bonferroni post-hoc test. Error bars correspond to S.E.

Previously, we identified a mutant allele for *FIP1* gene, *fi1p-2*, that showed defects in polyadenylation site usage and selection, formation of LRs and altered expression of nitrate transporters [12]. Given the central role that roots play in nitrogen nutrition, we studied the responses to N starvation in the wild-type and *fip1-2* mutant. N starvation did not reduce root growth in *fip1-2* compared with control seedlings (Figure 1A,B). As root illumination has a negative effect on root growth during nutrient deficiency conditions [18,19,20], we decided to analyze the effect of root illumination in a medium containing different concentrations of N. In control seedlings, decreasing the level of N reduced root growth, and this reduction was significantly higher in light grown-root seedlings (LGR) than dark-grown root seedlings (DGR) (Figure 1C), showing an interaction between light and N deprivation. However, these differences in root growth were not observed in *fip1-2* mutant (Figure 1C), that was only influenced by the total lack of N in the medium, but not by the light. In addition, the expression of NRT2.4::GUS marker, which is induced by N deprivation [7], was analyzed. As shown in Figure 1D,E, GUS activity was induced in control roots when seedlings were grown in a medium containing a low amount of N (50 µM), but the GUS staining was clearly reduced in *fip1-2* mutant, either in DGR or LGR seedlings. We also found that the expression level of other N deficiency response regulators was reduced in *fip1-2* roots (Figure S1A). Interestingly, the expression of *NRT1.1* and *AFB3*, two key components of the nitrate signaling [21], were down-regulated in *fip1-2*. In addition, the larger transcript isoform of *CPSF30-L*, which seems to have an important role in nitrate signaling, did not change its expression level, while the shorter isoform was down-regulated.

We also analyzed the effect of low N in the formation of emerged LRs (eLR) using the SKP2B::GUS, a well-defined root formation marker [22]. In control seedlings, N levels ranging from 1000 µM to 20 µM decreased the number of total eLR by about 35% compared with plants grown on 2.5 mM of N, while eLR production on plants grown without N was about 18% of that seen in plants given 2.5 mM N (Figure 1F). In the *fip1-2* mutant, eLR numbers on plants grown in 100 µM or 20 µM N were indistinguishable from those seen in the absence of N (Figure 1F). According with this molecular phenotype, we found that *fip1-2* mutant accumulated significantly less nitrate than control seedlings (Figure 1G). We also found that light had a minor impact on nitrate accumulation when seedlings were grown with high N, but in a low N medium, root illumination reduced the accumulation of NO_3_^−^ by 60% or 36% in control seedlings and *fip1-2* mutant respectively (Figure 1G). An element analysis showed that, in general, total ions content was rather similar between control and *fip1-2* seedlings. However, significant lower content for several micronutrients such as Ca, Mg, Mn, Na, Sr, Ni or Cr and slightly higher levels of Fe was detected in *fip1-2* compared with control seedlings (Figure S2), suggesting that specific ion absorption might be modified in the mutant. Taken together, our data indicate that *fip1-2* mutant was insensitive to the negative effect of combining light and N deprivation. As light seems to have an additive effect on N starvation response, subsequent analyses of the root system were done using DGR seedlings.

### 2.2. FIP1 Regulates Alternative Polyadenylation in Response to Low Nitrogen

**Figure 2:**
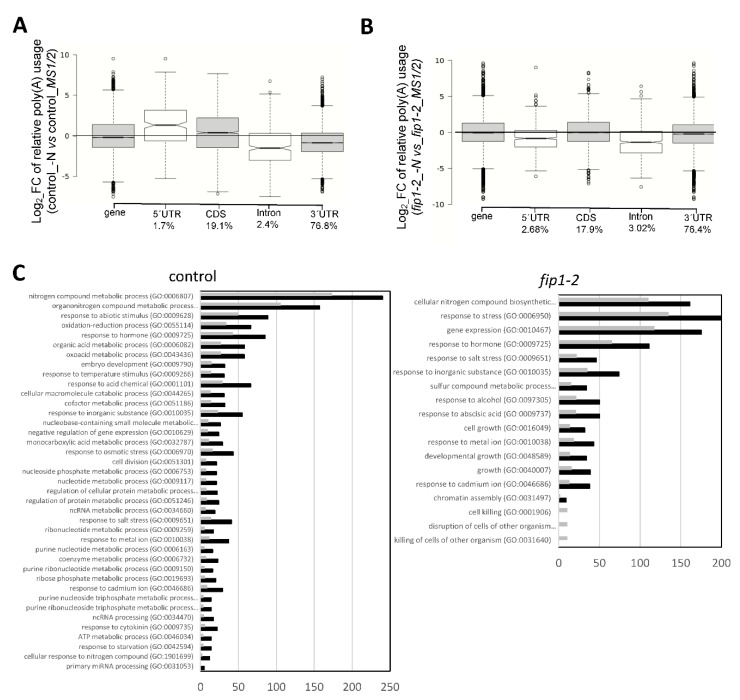
Genome-wide distribution of poly(A) site usage in response to nitrogen starvation and mediated by FIP1. Box plots showing change in usage of different classes of poly(A) sites. For this, the relative contribution that each poly(A) cluster (PAC) makes to total poly(A) usage was determined on a gene-by-gene basis, with the ratios of usage in nitrogen starved roots in control seedlings (**A**) or in *fip1-2* (**B**) calculated and log2-transformed. “Gene” represents the complete collection of PACs analyzed. Bottom percentage indicates the relative number of PACs in each locus position. (**C**) Gene ontology analyses of genes showing APA in control or *fip1-2* in response to N deficiency in roots with FDR <0.05 and a fold enrichment >2. Grey bars correspond to expected number of genes and black bars to observe number. For full list of GO categories, see Table S1.

As *FIP1* regulates polyadenylation site usage in response to environmental changes [12], we decided to analyze the global effect of N deficiency on poly(A) site selection. Genome wide analyses showed that most poly(A) sites were localized in the 3′-UTRs of the affected transcription units (Figure 2A). In control seedlings, the levels of mRNA isoforms with poly(A) 3′-ends inside of coding regions or 5′-UTRs increased upon N starvation, while isoforms with poly(A) into introns decreased (Figure 2A). In contrast, in the *fip1-2* mutant, there was no corresponding increase in the levels of mRNAs ending within coding regions or 5′-UTRs under N-deficient conditions (Figure 2B). There was also no apparent large-scale remodeling of canonical poly(A) site choice in the mutant (“3′-UTR” in Figure 2B). The levels of mRNAs ending within introns decreased in N-deficient conditions in the *fip1-2* mutant compared with control seedlings. These results suggest that *FIP1* may be directly responsible for the N deficiency-associated changes in poly(A) site choice.

Using the DEXseq package [23], we determined that these poly(A) changes in response to N deficiency affected 707 different loci in control roots and over 1000 loci in *fip1-2* roots (Table S1). Gene ontology analyses revealed that N starvation in control roots led to APA in genes involved in response to biotic and abiotic stresses, response to N compounds metabolism, ATP metabolic processes, response to hormones, or response to salt among others (Figure 2C, Table S1B). In the case of *fip1-2*, APA induced by N starvation affected genes involved in root system development, growth, chromatin assembly, responses to biotic and abiotic stresses, hormonal responses, and N-based metabolism among others (Figure 2C, Table S1D). In both genetic backgrounds, APA affected a large number of transcripts related to nitrogen metabolism (Table S1G).

**Figure 3:**
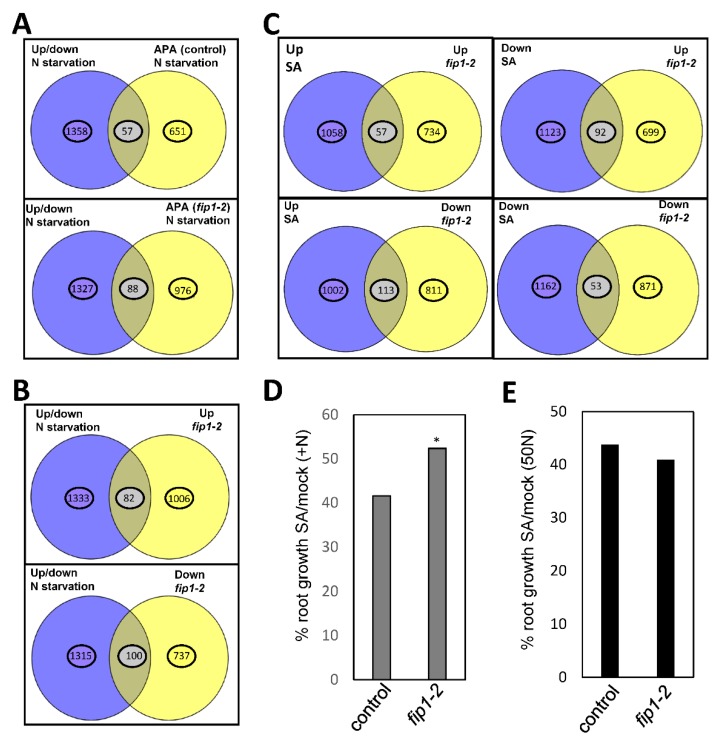
Transcript overlapping between alternative polyadenylation and N starvation responses and salicylic acid response. (**A**) Venny´s diagrams of common genes between of those up- and down-regulated (Up/down) by nitrogen starvation and transcripts showing APA in control or *fip1-2* (**B**) Venny’s diagrams of common genes between genes up- and down-regulated by nitrogen starvation and genes up-regulated (Up) or down-regulated (Down) in *fip1-2*. (**C**) Venny’s diagrams of common genes between genes up-regulated (Up) or down-regulated (Down) by salicylic acid treatment and genes up-regulated (Up) or down-regulated (Down) in *fip1-2*. The full list of overlapping genes can be found in Table S2. (**D**,**E**) Percentage of main root growth of seedlings grown for 5 days in MS1/2 medium with high N (+N) (D) or low N (50N) (E) and then 5 days to a similar medium containing 0 or 50 µM of SA. *, *p* < 0.05 by *t*-Test compared to control.

Next, we analyzed the overlapping between genes whose expression is altered in Arabidopsis roots by N starvation [24] and those that showed differentially poly(A) usage in response to N starvation (this work). Genes whose expression was statistically significant higher than 2-fold or more and lower than −2-fold were identified and overlapped with the sets of genes affected by APA upon N deprivation. We found that between 3 and 4% of the genes in each class were common (Figure 3A,B and Table S2A), both in control and *fip1-2* plants, respectively. This result indicates that, for the most part, APA affects a set of genes that are somewhat distinct from those whose overall expression changes in response to N starvation, in both genotypes.

Recent reports showed that *FIP1*, as well as *CPSF30*, is also involved in nitrate response [16,17]. Specifically, the larger of the two CPSF30 isoforms (CPSF30-L) was found to be necessary for nitrate-responsive transcription, such that mutant plants that express only the shorter CPSF30 isoform (CPSF30-S) lacked the nitrate-responsive expression of a reporter [17]. Inspection of the PATSeq data confirmed that both isoforms of CPSF30 were expressed in control seedlings grown under N-sufficient conditions (Figure S1B). However, in *fip1-2* or in N-starved control seedlings, only transcripts encoding a polyadenylated *CPSF30-L* isoform were apparent (Figure S1B).

Using an RT-PCR assay, greater usage of a promoter-proximal poly(A) site in transcripts encoding NTR1.1 was seen in the fip1 mutant [16]. Inspection of our PATSeq data also showed greater usage of the shorter (promoter-proximal) poly(A) site in the fip1-2 mutant (Figure S1B). Changes in poly(A) site choice in other N-regulated genes was also seen in the fip1-2 mutant (Figure S1C). These results corroborate and extend earlier reports and reveal specific effects of the fip1-2 mutation on the expression of N-regulated genes.

### 2.3. Hormonal Effect on Nitrogen-Starved Dark Grown Roots Seedlings

**Figure 4:**
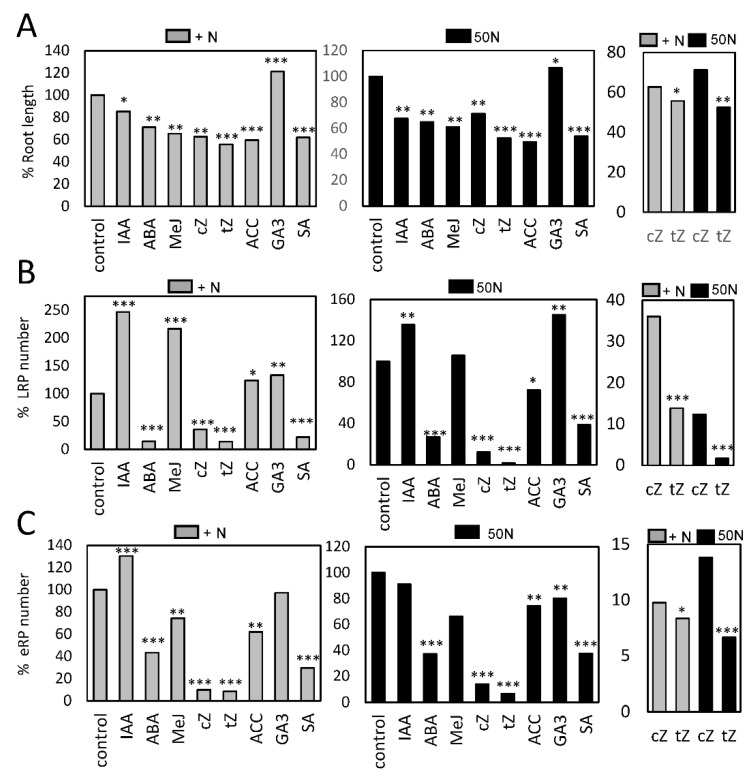
Effect of phytohormones on root growth and LR formation during N starvation. Dark-root growth Arabidopsis seedlings were grown with high (grey boxes) or low (black boxes) nitrogen for 5 days and then transferred to fresh medium containing similar N content and different hormones for 5 more days. Root length (**A**), lateral root primordia (LRP) (**B**) and eLRs (**C**) were quantified. Graphs represent the percentage of hormone-treated seedlings in respect to non-treated. *, *p* < 0.05; **, *p* < 0.01; ***, *p* < 0.001 by *t*-test compared to the respective control (mock) and nitrogen level. Right graphs show a magnification of the cZ and tZ effects. In this case, the significance was analyzed by t-test comparing the effect between cZ and tZ.

Phytohormones play important roles in the responses to nutritional deficiencies, including N deficiency [20,25,26]. To analyze the interplay between hormones and N deficiency in roots, we grew NRT2.4::GUS seedlings in mediums with high (2500 µM) or low (50 µM) N and then they were treated with different hormones. Subsequently, root length, LR formation and *NRT2.4* expression were studied. We found that, in general, all hormones tested, except gibberellin (GA3), significantly reduced root growth during N starvation (Figure 4A). We also found that the positive effect of methyl jasmonate (MeJA) on LR primordia formation was reduced during N starvation, while GA3 significantly increased the number of LRP in both conditions (Figure 4B). A significant reduction of LRP was also observed when abscisic acid (ABA), cisZeatins (cZ), transZeatins (tZ) and SA were added either to a medium with high or low N. It is noteworthy that only in the case of SA, the relative number of LRP was higher in seedlings grown in low N than with high N. All the hormones tested, except auxin (IAA) and GA3, reduced the emergence of LR, suggesting that some hormones, such as the ethylene precursor 1-aminocyclopropane-1-carboxylic acid (ACC) or MeJA, favored the specification of LRP but prevented the emergence of these primordia during N starvation.

The tZ isomer seems to have a specific and important role in shoot apical meristem during nitrate signaling [27]. Recently, we have found that cZ/tZ balance has an important role during the response to Pi starvation [20]. Similarly, we found that root growth inhibition and reduction of LRP and emergence of LRs was significantly different between cZ and tZ, especially when seedlings were grown in a low N content medium (Figure 4), suggesting that cZ might have specific roles during N starvation as well.

**Figure 5:**
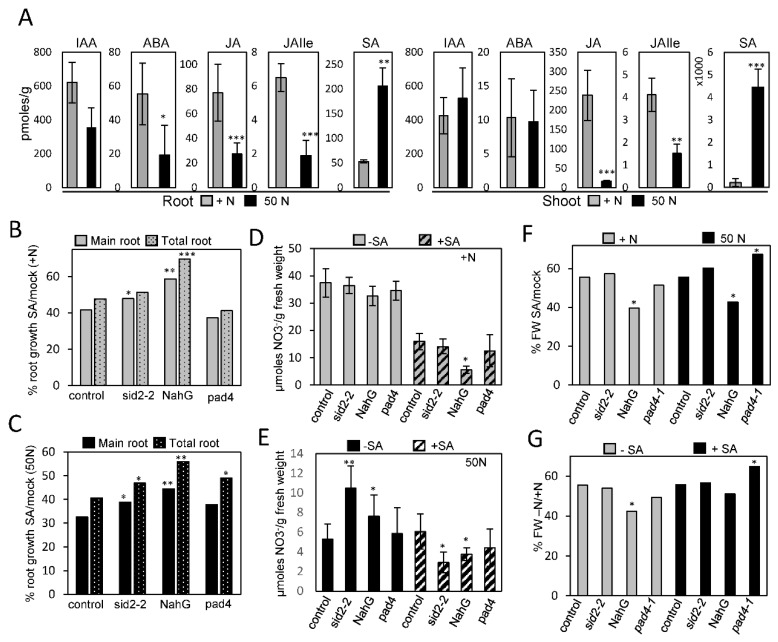
Effect of phytohormones in N starvation responses on roots. (**A**) Relative levels of Indol acetic acid (IAA), Abscisic acid (ABA), Jasmonic acid (JA), Jasmonic isolecuine (JAIle) and Salicylic acid (SA) in root or shoot of DGR seedlings that were cultivated in MS1/2 with high N (+N; 2500 µM) or with low N medium (50N; 50 µM) during 8 days. *, *p* < 0.05; **, *p* < 0.01; ***, *p* < 0.001 by *t*-Test comparing between high and low N. (**B**,**C**) Percentage of root growth (main root or main + LR length) of seedlings grown for 5 days in MS1/2 medium with high (2500 µM) (B) or low (50 µM) N (C) and then 5 days to a similar medium containing 0 or 50 µM of SA. *, *p* < 0.05; **, *p* < 0.01; ***, *p* < 0.001 by *t*-Test comparing to the control. (**D**,**E**) NO_3_^−^ levels in control seedlings grown in MS1/2 medium containing high N (2500 µM) (D) or low N (50 µM) (E) during 5 days and then transferred to a similar medium containing 0 or 50 µM of SA for 5 extra days. *, *p* < 0.05; **, *p* < 0.01; ***, *p* < 0.001 by *t*-Test comparing to the control in each condition. (**F**) Relative fresh weight (FW) of seedlings treated with or without SA that were grown as in (D). (**G**) Relative fresh weight (FW) of seedlings grown with or without N that were grown as in (E). *, *p* < 0.05; **, *p* < 0.01; ***, *p* < 0.001 by *t*-Test comparing to the control in each condition. Error bars correspond to S.E.

NRT2.4::GUS seedlings were grown with a low amount of N (50 µM) to induce the marker and then treated with different hormones. We found that ABA treatment completely abolished the expression of *NRT2.4* gene in the entire root system compared with the mock (Figure S3A,B), while cZ and tZ suppressed the expression in the main root tip and in LRs, except in the LR tip (Figure S3C,D). MeJA treatment only eliminated the expression of *NRT4.2* from the main root (Figure S3E). Conversely, IAA slightly increased GUS staining in the root tip and LR (Figure S3F). The ethylene precursor ACC increased GUS staining in LRs (Figure S3G) while GA3 decreased the GUS staining in the root tip (Figure S3H) and salicylic acid (SA) did not significantly affect GUS staining (Figure S3I). These data suggest that hormonal signaling might be important in the response to N starvation. Therefore, we decided to quantify the levels of different hormones in response to N deficiency. We found that IAA levels did not significantly change in either root or shoot, although there was high variability between samples (Figure 5A). ABA levels slightly decreased in roots while jasmonic acid (JA) and jasmonic acid isoleucine (JAIle) levels significantly decreased in both roots and shoots in response to N scarcity (Figure 5A). Conversely, SA levels significantly increased in both roots and shoots in response to low N (Figure 5A).

The significant increase in SA prompted us to analyze its role during the response to N starvation and the possible connection with FIP1-mediated responses. First, we found a statistically significant overlapping between SA de-regulated genes [28] and those genes de-regulated in *fip1-2* [12] (Figure 3C and Table S2B). We also found that *fip1-2* was slightly, but significantly more resistant to SA-mediated root growth inhibition in a medium containing high N (Figure 3D). However, this resistance to SA was abolished when they were grown in a medium with low amount of N (Figure 3E), and although not statistically significant, in these conditions, *fip1-2* seems to be more sensitive to SA. Next, we quantified the main root growth and total root growth (main plus LRs length) in response to low N and SA treatment in control, *sid2-2* mutant and NahG lines, which accumulated lower level of SA [29,30], and the *pad4-1* mutant, a lipase important for SA accumulation upon pathogen infection [31]. We found that treatment with 50 µM SA reduced root growth (main root or total root length) in all genotypes, but this reduction was significantly minor in *sid2* and NahG (Figure 5B). A similar trend was observed when seedlings were grown with low N, except for *sid2* and *pad4*, which increased total root length (Figure 5C). This result is in agreement with the proposed role of SA in reducing the main root and LR growth [32]. We also tested the role of SA in nitrate (NO_3_^−^) accumulation. As shown in Figure 5D, SA treatment reduced the level of NO_3_^−^ in control seedlings and to a larger extend in NahG lines. In low N conditions, NO_3_^−^ levels were significantly higher in *sid2* and NahG (Figure 5E). However, this trend changed when seedlings were grown with low N and treated with SA, as *sid2* and NahG reduced the amount of NO_3_^−^. Finally, we also observed that NahG showed a reduction in plant growth, which was measured as fresh weight, in response to SA or N deficiency (Figure 5F,G).

We analyzed the poly(A) usage in key components of SA biosynthesis, perception or signaling [33,34]. We found that N starvation and/or *fip1-2* mutation altered the poly(A) usage in *NPR3*, a member of the SA receptor family, in *CAMTA2* and *CAMTA 3*, which function as repressors of SA signaling or *TGA5*, a cofactor of *NPR1* (Figure S4). In *NPR3*, N starvation reduced the poly(A) usage in the first exon, which might increase the functional level of this receptor and therefore SA signaling. In *CAMTA2* and *CAMTA3*, N starvation or *fip1-2* mutation increased poly(A) usage in a 3′-UTR proximal exon, which might reduce or inhibit their function. In the case of *TGA5*, N starvation almost abolished the poly(A) usage in different exons, which, in combination with the increase in SA levels, might enhance its positive function. A differential poly(A) usage was also found in *HY5*, a mobile signal that coordinates light-photosynthesis, N assimilation [34] and EDS1-dependent SA signaling [35]. There is an increased poly(A) usage in the third exon in response to N starvation that might reduce its activity.

We also analyzed the overlapping between genes whose expression is altered in Arabidopsis roots by N starvation [24] and those altered by SA treatment [28]. We only found statistical overlapping between up-regulated genes by SA treatment and N starvation response or between down-regulated genes by SA treatment and N starvation response (Figure S5), suggesting a link between both responses. Gene ontology analyses showed that the common genes were enriched in responses to abiotic and biotic stresses, oxygen-containing compounds, and photosynthesis (Figure S5).

## 3. Discussion

Responses to low N involve changes in gene transcription that modulate different pathways. Some pieces of evidence point out that APA regulates nitrate signaling [16,17]. In this work, we show global polyadenylation usage changes in response to N deficiency and the role of *FIP1*, a core poly(A) complex subunit, in this response. Specifically, similar to hypoxia or elevated levels of NaCl [12,14], upon N deprivation, the levels of mRNA isoforms with poly(A) tag within 5′-UTRs increase substantially, and those with poly(A) within protein-coding regions increase more modestly (Figure 2A). These changes are not seen in the *fip1-2* mutant subjected to N deprivation, implicating FIP1 activity in the increased 5′-UTR and CDS poly(A) site usage. FIP1 has also been involved in the stress-associated increase in usage of polyadenylation in non-canonical regions in plants subjected to elevated salinity [12]. Taken together, these results place FIP1 in a regulatory pathway that connects stress signaling with APA and suggest that some aspects of the differential usage of sites that lie within 5′-UTRs and protein-coding regions are directly controlled by FIP1. More significantly, the relative insensitiveness of the *fip1-2* mutant to N starvation (Figure 1) and elevated salinity [12] suggest that the FIP1-dependent increase in non-canonical polyadenylation is one mechanism by which stresses might inhibit overall plant growth. This effect should take place in the root apical and LR meristems, as *FIP1* is only expressed in these areas during N starvation (data not shown).

In Arabidopsis, two transcripts are encoded by the *CPSF30* gene, a smaller one (CPSF30-S) and a larger one (CPSF30-L) [36]. CPSF30-L, which interacts with FIP1 [16], functions in the *NRT1.1*-nitrate signaling pathway to help nitrate uptake and assimilation [17]. At this time, it is difficult to determine the connection (if any) between the FIP1-dependent production of CPSF-S and N-responsive gene expression. It may be that regulation of the ratio of CPSF30-S/CPSF30-L, which seems to be dependent on FIP1 activity, is important for the functioning of CPSF30-L in N-responsive gene expression [16]. This possibility would add mechanistic detail to the roles played by FIP1 during N starvation. Our genomic analyses reveal that N starvation triggers a wide-ranging degree of APA. Remarkably, a significant number of the affected genes are associated with N signaling and N-metabolism, and also with hormonal signaling. Thus, our data represent a significant advance to understand the role of APA and *FIP1* function in the response to N starvation and plant adaptation to environmental changes.

Plant hormones play important roles in the responses to nutritional deficiencies [26]. Cytokinin seems to act as a local and systemic signal to coordinate the demand and acquisition of N. The pharmacological application of cytokinin represses *AtNRT2* genes, including *NRT2.4*, which are induced during N deficiency in roots [25]. Among the different cytokinins, tZ seems to have an important role in the control of stem cell activity in the shoot apical meristem in response to nitrate levels [27], while cZ plays an important role during phosphate starvation [20]. Our data show that both isomers cZ and tZ abolish the expression of *NRT2.4* in the root tip but not in LRs, suggesting that zeatins might favor N acquisition from a shallow-lateral root system. In addition, ABA, a hormone that blocks LR formation [37], completely eliminates *NRT2.4* expression in roots and reduces LRP formation. This negative effect of ABA correlates with a reduction of ABA levels in N starved roots, likely to allow LR formation and development to search for additional N sources in the medium.

The function of SA in pathogen responses has been well studied [38]. However, the knowledge of SA role in plant development and adaptation to abiotic stresses is limited. Several reports have revealed roles for SA in plant responses to abiotic stresses such as drought, chilling, heavy metal toxicity, heat, and osmotic stress [39], leading to the proposal of SA as a “health-supervisor hormone” for plants. SA has also been implicated in plant nutrition and yield [40,41,42]. Studies in rice show that foliar application of SA did not mitigate the negative effect of N starvation stress but reduces N content [42]. Our data suggest a link between SA responses and the plant response to N starvation. First, during N starvation, SA accumulates substantially in shoots and, to a lesser extent, in roots. Second, NahG lines and *sid2* mutants, which increase seed yield in Arabidopsis but slightly decrease N content [43], respond to N starvation differently than control seedlings. Finally, SA treatment significantly reduces NO_3_^−^ content but not the fresh weight in young Arabidopsis seedlings. This suggests that SA might favor the usage of free NO_3_^−^ to alleviate the stress caused by N starvation. This idea is supported by different reports that showed that SA alleviates abiotic and biotic stresses by increasing photosynthesis and growth [41]. It is possible that N-starved plants increase SA levels to balance plant growth with N levels. Alternatively, it is possible that SA acts locally in the roots to prevent overgrowth in areas where this nutrient is not available, allocating the resources in N-rich patches. Further analyses will help to clarify the precise role of SA in nitrogen starvation response. Furthermore, this work establishes a novel interplay between APA, nitrogen nutrition, and SA signaling. We think that N starvation triggers FIP1-mediated differential poly(A) usage that might induce changes in SA signaling (Figure S4). In addition, SA and N starvation responses are interconnected, as both share a significant number of common genes that are involved in abiotic and biotic stresses as well as in photosynthesis (Figure S5). As N is a major component for the photosynthetic machinery, it is possible that one of the responses of N deficiency is to increase SA levels to represses the photosynthetic capacity. *HY5*, which connects light-photosynthesis with N assimilation, induces the nitrogen transporter *NRT2.1* in roots. Thus, it is possible that, in an environment lacking N, *HY5* is not needed. Therefore, although speculative, it is possible that in response to N starvation, *HY5* accumulates non-canonical polyadenylation in an exon, an event that is dependent on *FIP1*, to reduce its function. Further analyses will be needed to confirm this possibility.

## 4. Conclusions

In this work, we present data showing that alternative polyadenylation and salicylic acid signaling act together during the response to N starvation. Several works showed that N starvation induces many changes in the plant transcriptome in response to this nutrient deficiency. However, changes in poly(A) usage to increase the transcriptome also seems to play an important role in such adaptation. Interestingly, and similar to other abiotic stresses responses, N starvation increases the polyadenylation in non-canonical sites of the mRNA in an FIP1-dependent manner. In fact, a mutation in this gene (*fip1-2*) affects the response of the Arabidopsis seedlings to N starvation. Finally, our data show that the salicylic acid, whose levels increase during N starvation, plays important roles during this nutritional deficiency in modulating the root system growth.

## 5. Material and Methods

### 5.1. Plant Material

Control seedlings (*SKP2Bp*:GUS) and *fip1-2* allele [22], NahG [29], *sid2* mutant [30], and pad4-1 [44] were used. We generated the NRT2.4::GUS line by cloning 2.4 Kb promoter region of the *NTR2.4* gene into the pGWB3 vector [45]. Arabidopsis plants (Columbia Col-0 ecotype) were transformed with this construct using the agroinfiltration method by means of *Agrobacterium tumefaciens C5801* [46].

All seedlings were sown under sterile conditions on vertically-oriented 12 cm square plates containing half-strength Murashige and Skoog (MS1/2), 0.05% MES, 1% sucrose and 1% plant-agar (Duchefa Biochemie B.V., 2003 RV Haarlem The Netherlands). For those experiments without nitrogen, the medium was similar but no source of nitrogen was added and K levels were compensated with KCl. To prepare MS1/2 with different concentrations of N, we added KNO_3_:HN_4_NO_3_ in a 55%:45% proportion and adjusted with KCl up to 9.4 mM. The medium named as high N contains 2500 µM of N (1375 µM of KNO_3_ and 1125 µM HN_4_NO_3_) and the medium named low N contains 50 µM N (27.5 µM of KNO_3_ and 22.5 µM HN_4_NO_3_,). The medium –N (without N, contains 0 µM of N). For SA treatments, seedlings were grown for 5 days in high or low N and then transferred to similar medium containing 50 µM of SA for the indicated days.

### 5.2. Root Growth Assays, GUS Staining and Microscopic Analysis

When indicated, seedlings were cultivated with the D-Root system to maintain the root system in darkness [19]. Primary root length was determined as described previously by [47] in half-strength MS (MS1/2) medium with N or different concentration of N as indicated for 10 days. All data are the mean value of at least 50 plants, and these experiments were repeated twice, obtaining similar values in each experiment. To measure the number of LRP, we used the SKP2B::GUS line [22] that was grown in MS1/2 with N or different concentration of N as indicated for 10 days. This line was stained for GUS activity in the growing plate for 16 h, adding the β-gluconidase substrate in 50 mM phosphate buffer pH = 7 with 0.02% of acetone. This staining maintains the root system architecture to facilitate the LRP and emerged LR counting. Pictures were taken using a Leica stereomicroscope MZ9.5 with a DCF280 camera or a Leica MD2000 microscope with a DCF300 camera.

To analyze the effect of hormones in the root response to N starvation, SKP2B::GUS or NRT2.4::GUS seedlings were grown in MS1/2 or MS1/2 containing 50 µM of N for 5 days and then transferred to similar medium containing mock (DMSO) or 10 nM of Indol-Acetic Acid (IAA), 10 µM of Gibberellic acid (GA_3_), 5 µM of Abscisic acid (ABA), 2.5 µM of cisZeatin (cZ), 2.5 µM of transZeatin (tZ), 5 µM of 1-aminocyclopropane-1-carboxylic acid (ACC), 5 µM of Jasmonic acid (JA) or 50 µM of Salicylic acid (SA) for 5 extra days. All hormones were purchased from Duchefa, except cZ and tZ that were purchased from OlChemIm Ltd, 770 10 Olomouc, Czech Republic. Afterwards, plates were scanned at high resolution (800 dpi) with a CANON perfection V600 scanner and root length was measured. For lateral root primordia (LRP), similar experiments were carried out using the SKP2B::GUS lines. After 5 days in hormone or mock treatment, seedlings were stained (see below) and GUS stained lateral LRP or eLRs were counted under a stereomicroscope Leica Z9. The NRT2.4::GUS line was grown in MS1/2 medium with N for 5 days and then transferred to MS1/2 with 50 µM of N for 2 days.

### 5.3. Poly(A) Tag Library Preparation, Sequencing and Analysis

Arabidopsis seedlings were grown for 9 days in MS1/2 or MS1/2 containing only 5 µM of N (5.5 µM KNO_3_, 4.5 µM NH_4_NO_3_ and adjusted with 10 mM KCl). Total RNA was isolated from Arabidopsis roots using the Trizol reagent and RNeasy columns (Qiagen, Str. 1, 40724 Hilden, Germany). Quantity and quality measurements were taken using a NanoDrop spectrophotometer (Thermo Scientific, Waltham, MA 02451, USA) and a BioAnalyzer (Santa Clara, CA 95051, USA). Poly(A) tags (PATs) were generated with 1 μg of total total RNA. Libraries preparation, sequencing and analysis were done as described in [14]. Aligned reads were visualized with IGV2.3.

### 5.4. Hormone and Ionome Quantification

Wild type (Columbia-0 ecotype) seedlings were grown for 10 days in MS1/2 or MS1/2 containing only 50 µM of N. The extraction and purification of hormones were carried out using the following method: 0.25 g of liquid N_2_frozen plant tissue was homogenized with 2.5 mL of precooled (−20 °C) methanol:water:HCOOH (90:9:1, *v*/*v*/*v*, with 2.5 mM Na-diethyldithiocarbamate) and 25 µL of a stock solution of 1000 ng mL^−1^ of deuterium-labelled internal standards in methanol. Hormones were extracted by shaking the samples during 60 min at 2000 rpm at room temperature and analyzed as described in [20].

Wild type Arabidopsis seedlings and *fip1-2* mutant were grown in in MS1/2 in the LGR for 8 days. Then, roots and shoots were collected together and were dried at 100 °C for 24–48 h in an air-oven. Sample digestions were carried out with 3 mL of HNO_3_ (Sigma-Aldrich Trace Metal grade, Sigma St. Louis, MO 63118, USA) at 100 °C in a termostatized bath for 4 h. Each sample was analyzed as described in [20].

### 5.5. Nitrate Quantification

Quantification of nitrate was done following the protocol reported in bioprotocols [48]. Arabidopsis seedlings (control, *fip1-2*, *sid2-2*, *pad4-1* and NahG) were grown for 5 days in high or low N MS1/2 medium and then transferred to a similar medium containing mock or 50 μM of SA for 5 more days. Afterwards, seedlings were harvested and processed as described in the bioprotocol. Two biological replicates of three samples each were analyzed. For each sample, two technical measures were recorded to check the accuracy, and the average of both measures was used.

### 5.6. Statistical Analyses

The data were statically analyzed by one-way or two-way ANOVA when indicated or *t*-test using GraphPad Prism5 software. For one-way ANOVA, the univariate analyses were performed with Tukey’s post hoc test. The post-hoc analysis for two-way ANOVA were done using the Bonferroni test.

## Supplementary Materials

The following are available online at https://www.mdpi.com/2223-7747/9/2/251/s1, Figure S1: Expression of nitrogen response genes, Figure S2: FIP1 regulates ion accumulation, Figure S3: Hormone effect on N starvation gene expression, Figure S4: Alternative PolyAdenylation of SA signaling factors, Figure S5: Common genes between SA treatment and N starvation response, Table S1: Genes modified by Alternative polyadenylation (APA), Table S2: Common genes showing APA in response to N starvation, altered expression in in *fip1-2* and or by salicylic acid treatment.
