# Supplementary material for: Alternative Polyadenylation and Salicylic Acid Modulate Root Responses to Low Nitrogen Availability
Source: Plants (Basel). 2020 Feb 16;9(2):251. doi: 10.3390/plants9020251 (PMC7076428; doi:10.3390/plants9020251)
Supplement: Supplementary file 1 [file plants-09-00251-s001.zip › plants-708913-supplementary-for conversion/plants-708913-supplementary-for conversion.docx]

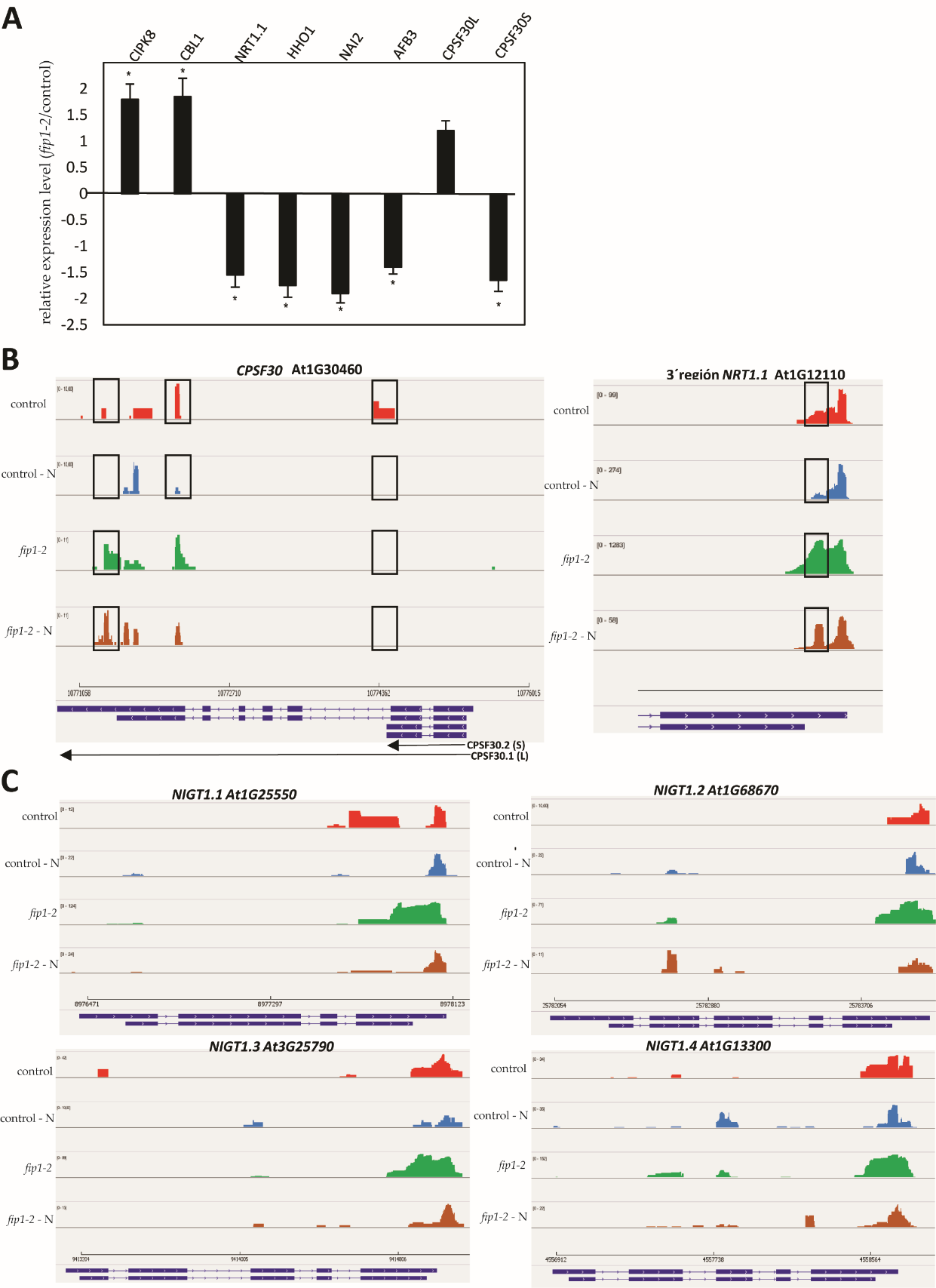


**Figure S1.** Expression of nitrogen response genes. (**A**) The expression of different nitrogen response genes were analyzed in 10 day-old control and *fip1-2* root s grown in MS1/2. The expression was analyzed by qRT-PCR as described in (Manzano et al., 2017). Statistically differences by a *t-Test*, * p < 0.05. Error bars correspond to S.E. (**B**) Screenshots of the polyadenylation pattern of *CPSF30* and *NRT1.1* loci. Rectangles indicate differences in the polyadenylation usage between control and *fip1-2* and in response to nitrogen. (**C**) Screenshots of the polyadenylation pattern of the four members of the *NIGT1* family involved in the response to N levels.

**
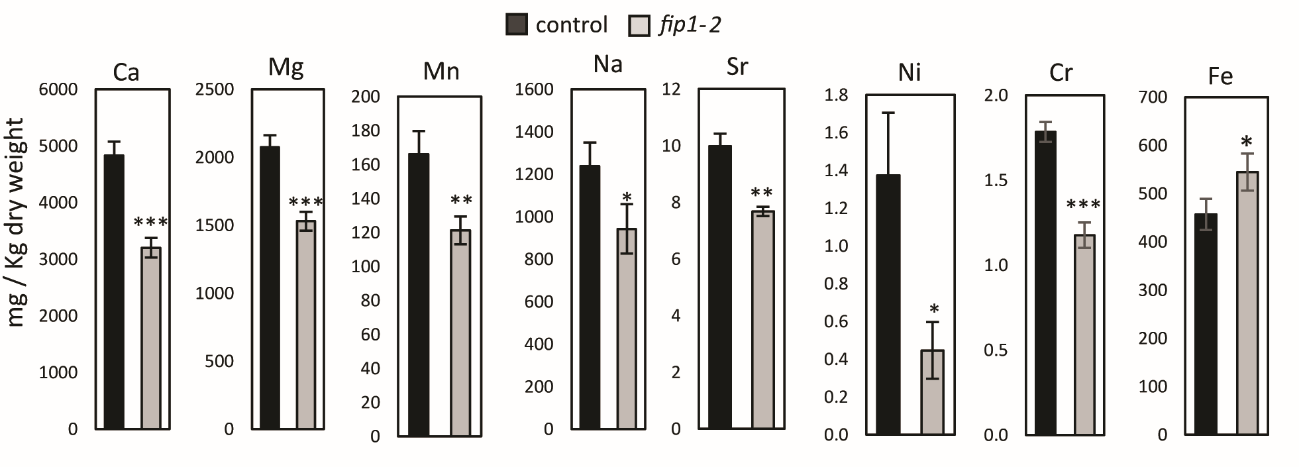
**

**Figure S2.** *FIP1* regulates ion accumulation. Significant changes in Ions accumulation in root of control and *ip1-2* seedlings. Graph represents elements in control (black) and *fip1-2* (grey). Data represents the average of 4 independent replicates of whole control or *fip1-2* seedlings grown for 12 days in MS1/2. Statistically differences by a *t-Test*, * p < 0.05; **, p < 0.01; ***, p < 0.001. Elements quantification was performed as described by Silva-Navas et al 2015, 2016.


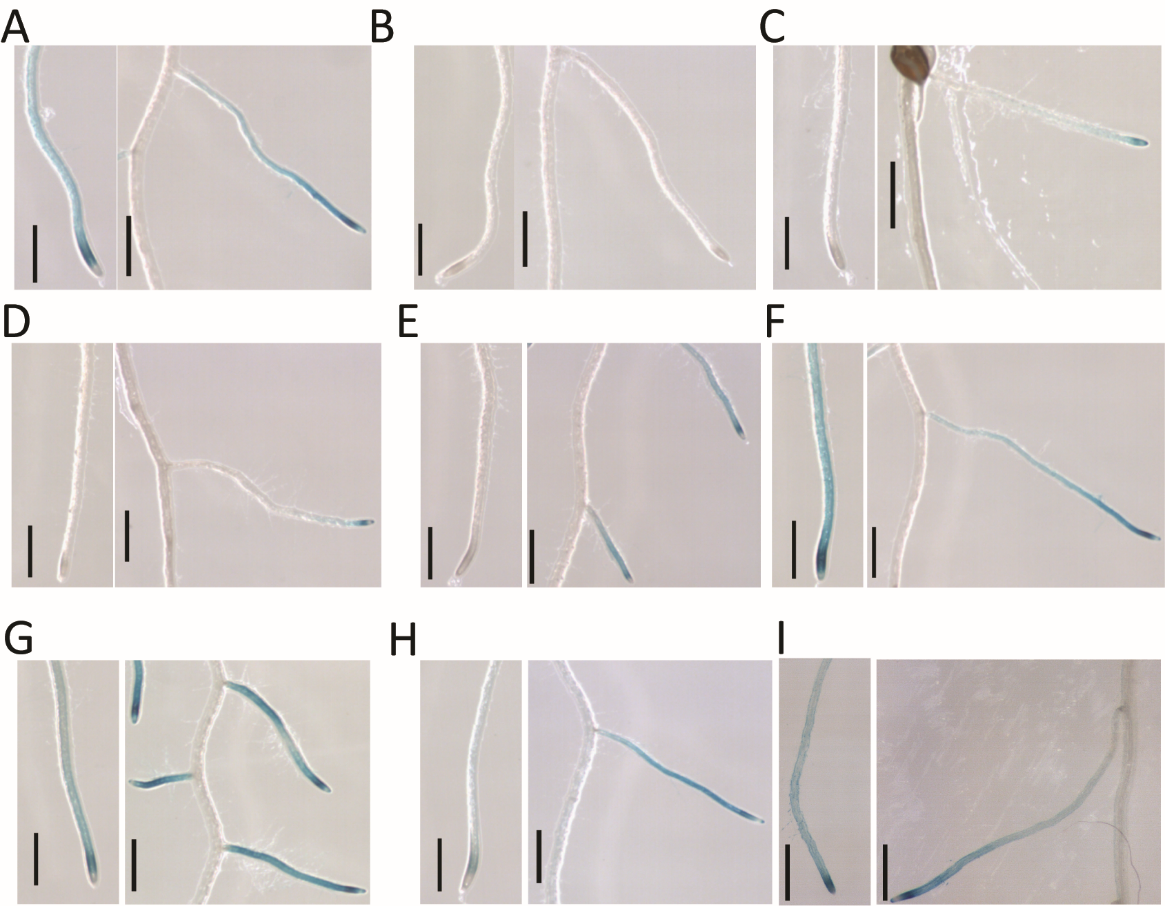


**Figure S3.** Hormone effect on N starvation gene expression. NRT2.4 seedling were cultivated in MS1/2 with or without nitrogen source for 5 days and then they were treated with different hormones for 5 extra days and stained for GUS activity in the plate. Stained roots were photographed with a Leica camera adapted to a stereomicroscope. **(A–I)** Dark-grown root NRT2.4::GUS seedlings were grown 5 days in MS1/2 medium with 50 µM of N and then transferred to a similar medium containing mock **(A)**, 5 µM of ABA (**B)**, 2.5 µM of cZ **(C)**, 2.5 µM tZ **(D)**, 5µM of MeJ **(E)**, 10 nM of IAA **(F)**, 5 µM of ACC **(G)**, 10 µM of GA3 **(H)** or 50 µM SA **(I)** for 5 extra days. ABA: Abscisic acid; cZ. CisZeatin; tZ: transZeatin; ACC: 1-Aminocyclopropane-1-carboxylic acid, ethylene precursor; MeJ: methyl Jamonate; IAA: Indole-Acetic acid; GA3 Gibberellin GA3; SA, salicylic acid. Scale bars= 400 µm.


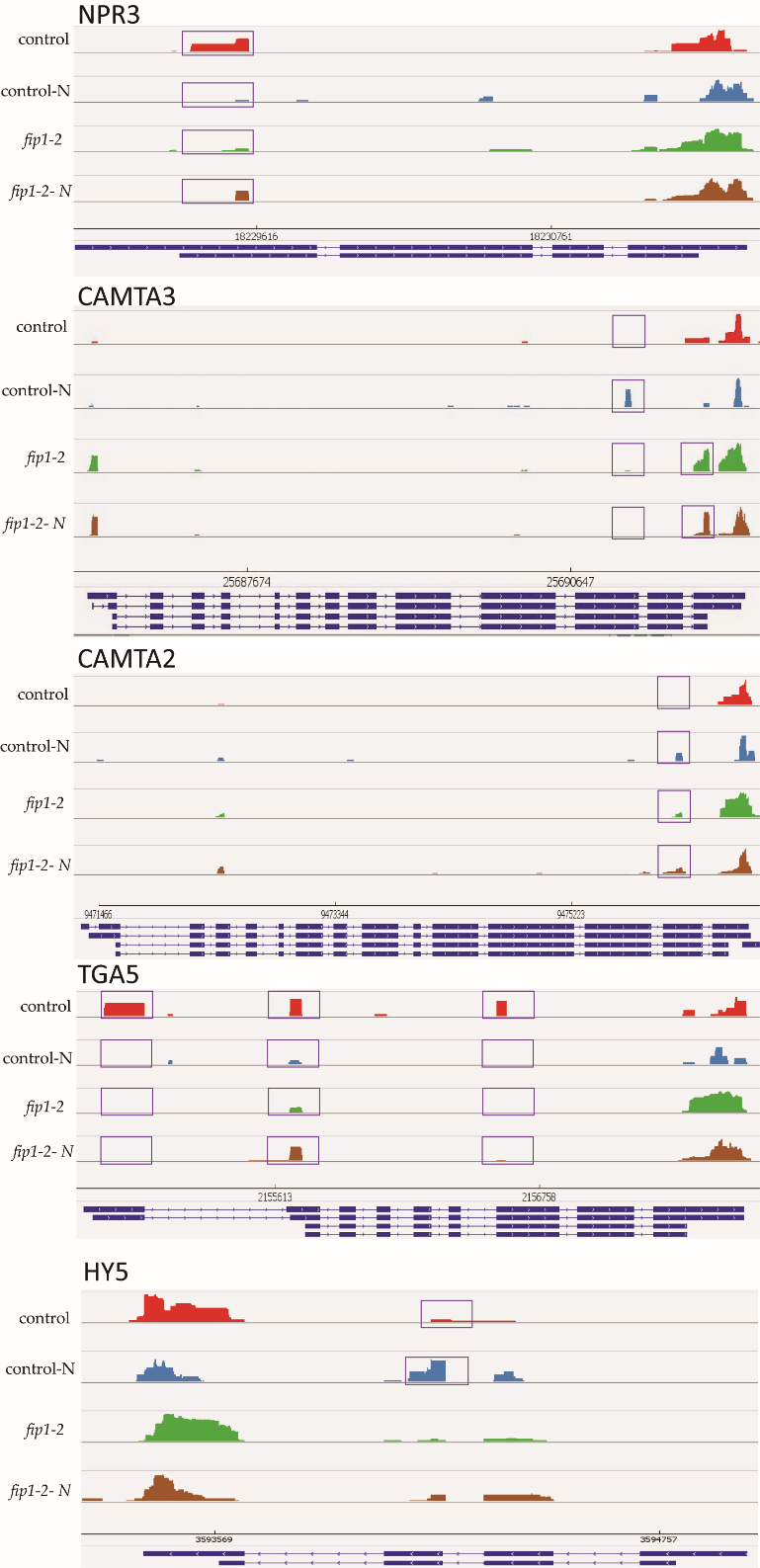


**Figure S4.** Alternative PolyAdenylation of SA signaling factors. Screenshots of the polyadenylation pattern of *NPR3, CAMTA3, CAMTA2, TGA5* and *HY5* mRNAs. Rectangles indicate differences in the polyadenylation usage between control and *fip1-2* and in response to nitrogen starvation. NPR3 is a member of the SA receptor family. CAMTA2 and 3 function as repressor of EDS1, which is a positive regulator of SA accumulation. TGA5 is a NPR1 receptor repressor. In the case of NPR3, N starvation reduced the poly(A) usage in the first exon, that might increase the functional level of this receptor and SA activity. In the case of CAMTAs, during N starvation or *fip1-2* mutant the poly(A) usage increased in the antepenultimate exon, that might reduce or inhibit their function. In the case of TGA5, the poly(A) usage in different exons is almost abolished during N starvation, that might increase its positive function in SA signaling. N starvation increased the poly(A) usage in the third exon of HY5 that might reduce HY5 activity.


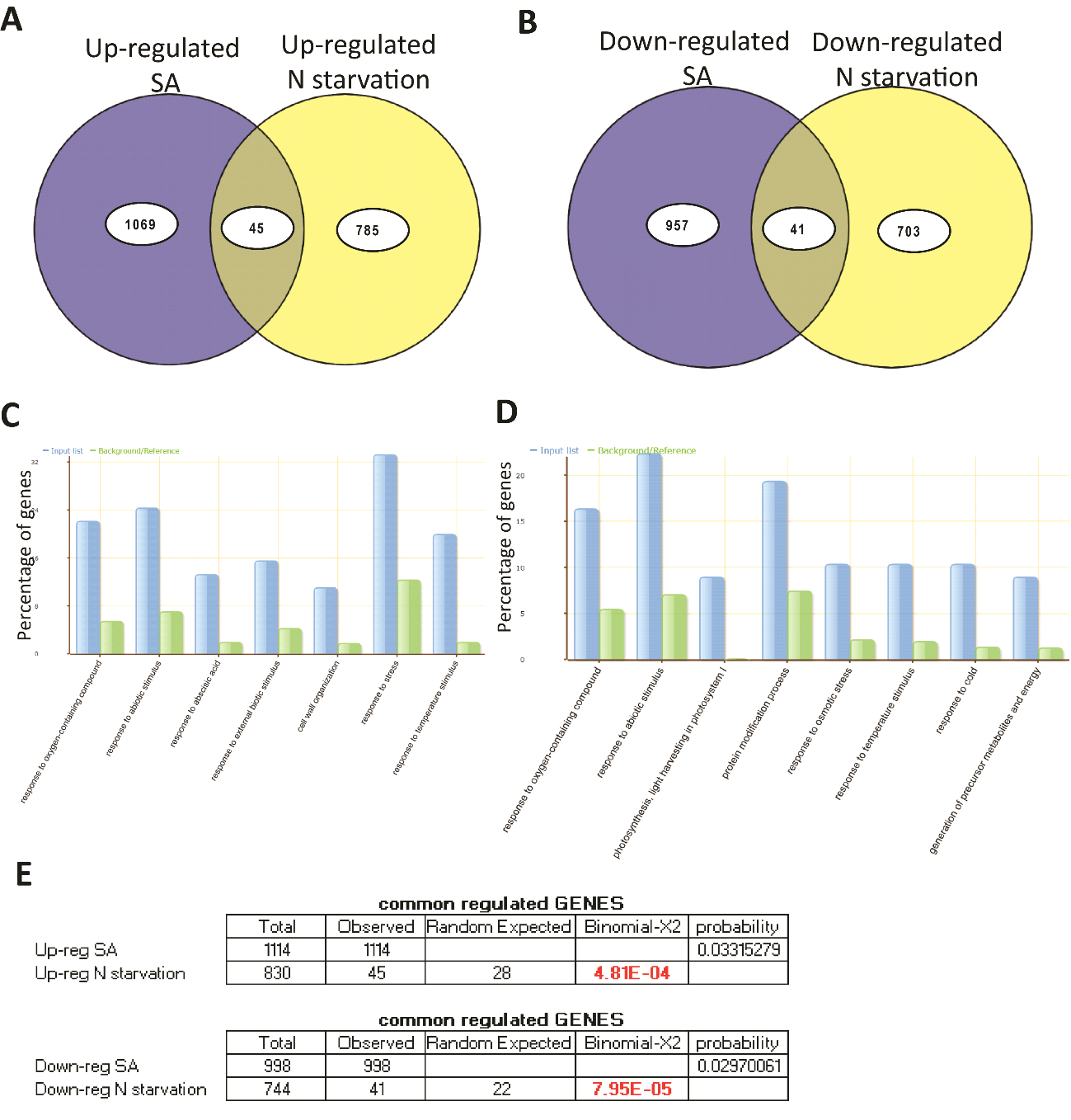


**Figure S5.** Common genes between SA treatment and N starvation response. (**A**) Venny´s diagrams showing SA treatment up-regulated genes or up-regulated genes that are induced by N starvation. (**B**) Venny´s diagrams showing SA treatment down-regulated genes and down-regulated genes that are repressed by N starvation. (**C**) Bar charts showing the most significant gene ontology categories of common genes in A. (**D**) Bar charts showing the most significant gene ontology categories of common genes in B. (**E**) The table indicates the number of overlapping genes up- or down-regulated by salicylic (SA) (Ding et al., 2018) and regulated by N starvation (Maeda et al., 2018). P-values for differences between observed and predicted random coincidences, were calculated using X2-test.
